# Acute appendicitis during pregnancy: a case-control study

**DOI:** 10.3389/fsurg.2026.1914944

**Published:** 2026-09-17

**Authors:** Bijian Liu, Changsheng Pu, Tao Wen, Chunbao Guo

**Affiliations:** 1 Department of Surgical Alliance, Maternal and Child Health System of Chongqing, Chongqing, China; 2 Department of Pediatric General Surgery, Hechuan Maternal and Child Health Hospital, Chongqing, China; 3 Department of Pediatric General Surgery, Chongqing Maternal and Child Health Hospital, Chongqing Medical University, Chongqing, China; 4 Department of Pediatric Surgery, Qijiang Health Center for Maternal and Child Care, Chongqing, China

**Keywords:** acute appendicitis, laparoscopic appendectomy, magnetic resonance imaging, pregnancy, ultrasonography

## Abstract

**Background:**

Acute appendicitis is the most common non-obstetric surgical emergency in pregnancy. Diagnosis is difficult due to atypical symptoms, anatomical changes, and limited imaging utility, while delayed diagnosis increases maternal-fetal risks. This study compared clinical, laboratory, imaging and perioperative outcomes between pregnant and non-pregnant women.

**Methods:**

This analysis included a multi-centered retrospective case-control study from July 1, 2020 to December 30, 2025. The operative characteristics involved in the pregnancy women were investigated using univariate analyses.

**Results:**

There were 99 pregnant women and 186 non-pregnant reproductive-age women underwent surgery enrolled in this research. Pregnant patients had lower right lower quadrant tenderness (*P* = 0.046) and lower ultrasound sensitivity (46.5% vs. 53.2%). MRI showed 100% diagnostic accuracy. Pregnant women had higher open conversion rate (*P* < 0.001), longer hospital stay (*P* = 0.0056) and higher histologically negative appendectomies (14.1% vs. 9.7%, *P* < 0.001). Laparoscopic rate decreased with gestational age. Most complications were minor.

**Conclusion:**

Acute appendicitis in pregnancy presents atypically with lower ultrasound sensitivity. MRI improves diagnostic accuracy. Laparoscopic appendectomy is safe and feasible in pregnancy.

## Introduction

Clinically suspected appendicitis is the most common non-obstetric surgical indication during pregnancy, with a reported incidence of 1 case per 500–2,000 pregnancies. Acute appendicitis can occur at any stage of pregnancy but is most common in the second trimester (1, 2).

Patients with acute appendicitis during pregnancy usually first present to obstetrics and gynecology departments, as pregnancy-related causes of abdominal pain are prioritized clinically. The diagnosis and management of these patients are highly challenging and require integration of clinical manifestations, laboratory tests, and imaging findings, often necessitating multidisciplinary collaboration between obstetricians and general surgeons. Delayed diagnosis is associated with increased maternal and fetal mortality and complication rates (3, 4).

Patients with acute appendicitis during pregnancy rarely present with classic clinical symptoms. Gastrointestinal symptoms and abdominal discomfort are common in normal pregnancy (5); in addition, the enlarged uterus alters anatomical position, leading to atypical pain location. Since leukocytosis can occur in healthy pregnant women, laboratory markers such as white blood cell count (WBC) and C-reactive protein (CRP) have limited diagnostic value. Mild leukocytosis with left shift is frequently observed in pregnancy (6–8).

The use of imaging in pregnant patients with appendicitis is limited by multiple constraints. The American College of Radiology (ACR) Appropriateness Criteria for suspected appendicitis in pregnancy recommend abdominal ultrasound as the first-line imaging modality. The main limitation of ultrasound is difficulty in visualizing the appendix, especially in the second and third trimesters (9). Computed tomography (CT) should be avoided due to radiation risk. Magnetic resonance imaging (MRI) has high diagnostic accuracy and is suitable for cases with inconclusive ultrasound results; it may also serve as a first-line imaging test. However, MRI availability is limited in many medical institutions, particularly at night. Even with negative or inconclusive MRI findings, surgical intervention should still be considered if appendicitis is highly suspected clinically (10). Due to insufficient evidence, non-operative management of uncomplicated acute appendicitis during pregnancy is not currently recommended, poorer clinical outcomes with non-operative treatment compared with appendectomy.

The use of laparoscopy in pregnancy has been controversial, but meta-analyses have confirmed that laparoscopic surgery is safe in terms of fetal loss and preterm birth risk. Therefore, laparoscopic appendectomy is preferred over open surgery during pregnancy when surgeon experience and equipment permit (11, 12).

Although acute appendicitis in pregnancy is relatively common, most existing literature consists of small-sample case series. This study aimed to compare the frequency and diagnostic accuracy of typical clinical signs, laboratory tests, and imaging findings between pregnant women undergoing surgery for suspected appendicitis and non-pregnant reproductive-age female controls, and to describe intra- and postoperative outcomes in both groups. As one of the largest single-center retrospective studies, it provides additional clinical evidence for the surgical management of appendicitis during pregnancy.

## Materials and methods

### Study population, definitions, and surgical techniques

A retrospective case-control study was conducted on female patients aged 16–49 years who underwent emergency surgery for suspected appendicitis at the Department of Surgery, Women and Children's Hospital of Chongqing Medical University, between July 1, 2020, and December 30, 2025. The case group included 99 pregnant women who underwent surgery during pregnancy; 186 non-pregnant reproductive-age women who underwent emergency appendectomy for appendicitis during the same period served as the control group. Patients with appendiceal abscess or diffuse peritonitis were included.

The study protocol was approved by the Ethics Committee of Chongqing Medical University, and the study was conducted in accordance with the ethical standards of the Declaration of Helsinki. After ethical approval, two independent investigators with specialized training comprehensively reviewed the medical records of all enrolled patients. All pregnant and non-pregnant females receiving emergency surgery due to clinically suspected acute appendicitis during the study period were enrolled. Exclusion criteria were confirmed tubo-ovarian abscess, acute pelvic inflammatory disease and pelvic congestion syndrome, and so on.

At our hospital, the diagnosis of appendicitis in pregnancy was made by the attending physician, based on initial physical examination, blood tests, abdominal ultrasound, and MRI when necessary, with obstetric causes excluded by an obstetrician. MRI was performed at the discretion of the on-call physician according to disease severity and equipment availability. The MRI examination was considered in pregnant patients when ultrasound findings were ambiguous (cannot rule out appendicitis), persistent right lower abdominal pain and inflammatory laboratory elevation, unclear clinical diagnosis after physical examination; ultrasound could not clearly display the appendix due to enlarged uterus compression. Non-pregnant patients mostly obtained definitive positive/negative appendicitis diagnosis via ultrasound; MRI was rarely arranged considering cost and unnecessary radiation-free imaging; only 1 non-pregnant patient received MRI when ultrasound was indeterminate.

All preoperative treatment plans were jointly evaluated by obstetricians and surgeons, and individualized according to patient conditions and the surgical team's preferences. Negative appendectomy was defined as postoperative pathological confirmation of no appendiceal inflammation.

Laparoscopic appendectomy was the standard procedure for non-pregnant women and patients in the first and second trimesters of pregnancy. No Veress needle was used in pregnant patients; the first trocar was inserted via a small supraumbilical incision. A Trendelenburg position with 10°–15° left lateral tilt was used for surgical access. The appendix was grasped with atraumatic forceps, and the uterus was gently retracted with atraumatic blunt instruments if needed.

Postoperative antibiotics were not universally administered. Antibiotic types, duration and dosage were individualized based on intraoperative pathological findings and disease severity: Histologically confirmed simple acute appendicitis without perforation or peritonitis: single-dose preoperative prophylactic antibiotics only, no postoperative antibiotics. Suppurative appendicitis: oral antibiotics for 2–3 days postoperatively. Perforated appendicitis, periappendiceal abscess or generalized peritonitis: intravenous broad-spectrum antibiotics piperacillintazobactam (100–150 mg/kg/day, twice daily) or cefoperazone sodiumsulbactam (100–150 mg/kg dose, twice daily) combined with metronidazole (30 mg/kg dose, twice daily) for 3–7 days, adjusted according to intraoperative pus culture results when available until serum CRP levels fell below 1.0 mg/dL. Patients with histologically normal appendix received only prophylactic single-dose preoperative antibiotics without postoperative antimicrobial treatment.

### Clinical data and outcome assessment

Perioperative data were extracted from electronic medical records, including age, ASA classification, BMI, clinical symptoms and physical examination findings on admission (onset and course of nauseavomiting, colic, abdominal distension, fever, bowel sounds), laboratory results (venous WBC, CRP if tested), original imaging data (B-ultrasound or MRI), surgery-related data (time from admission to surgery, intraoperative findings, operative duration, surgical approach [open or laparoscopic], laparoscopic rate, pathological results), and postoperative outcomes (intraoperative microbiological results, complication rate, length of hospital stay).

Systematic follow-up was conducted by surgeons via telephone at 1, 6, and 12 months postoperatively, and data on the date and type of obstruction-related treatment at our hospital or other surgical departments were collected. For patients admitted before December 1, 2025: clinical data and follow-up information at 1, 6, and 12 months after surgery were collected retrospectively from medical record system and telephone follow-up archives. For patients admitted between December 1 and December 30, 2025: basic clinical data were extracted retrospectively, and their 1-month, 6-month, 12-month follow-up was completed prospectively via telephone after discharge.

### Statistical analysis

Statistical comparisons were performed using SPSS 17.0 software (SPSS Inc., Chicago, IL, USA). Categorical variables were presented as frequencies (percentages), and continuous variables as mean ± standard deviation. Univariate analysis was performed using Student's t-test or Mann–Whitney U test for continuous variables, and Fisher's exact test or *χ*^2^ test for categorical variables. A two-sided *P* < 0.05 was considered statistically significant. Univariate analysis was used to compare demographic, clinical, laboratory, and imaging indicators between the two groups, as well as intra- and postoperative characteristics (e.g., pathological results, complication rates).

## Results

A total of 99 pregnant women and 186 non-pregnant reproductive-age women aged 16–49 years who underwent emergency surgery for suspected appendicitis were enrolled between July 1, 2020, and December 30, 2025. Among the 99 pregnant patients, 22 were in the first trimester, 47 in the second trimester, and 30 in the third trimester.

A comparative analysis of pregnant and non-pregnant patients undergoing emergency appendectomy was performed. There were no statistically significant differences in age, WBC, neutrophil count, neutrophil-to-lymphocyte ratio (NLR), or platelet-to-lymphocyte ratio (PLR) between groups (all *P* > 0.05). Mean age was 29.4 ± 6.4 years in the pregnant group and 28.6 ± 7.8 years in the non-pregnant group. Demographic characteristics and preoperative test results are summarized in Table 1. There were no significant differences in gender distribution, admission WBC, or CRP between groups (Table 1).

**Table 1:** Demographic and preoperative of eligible patients.

Characteristics	Pregnant (*n* = 99)	Non pregnant (*n* = 186)	*p* Values
Age (yrs)	29.4 ± 6.4	28.6 ± 7.8	0.35
BMI	26.8 ± 5.6	25.3 ± 5.5	0.31
Duration of clinical symptoms, days, Mean ± SD	2.1 ± 1.7	3.2 ± 2.1	0.062
Admission to surgery ≤ 12 hours	70 (70.7%)	133 (71.5%)	0.89
Abdominal pain	89 (89.9%)	178 (95.7%)	0.055
Nausea and/or vomiting	38 (38.4%)	76 (40.9%)	0.69
Fever	26 (26.3%)	57 (30.6%)	0.44
RLQ Tenderness	82 (82.8%)	169 (90.9%)	0.046
Pain migration	15 (15.2%)	38 (20.4%)	0.28
Guarding	22 (22.2%)	53 (28.5%)	0.25
Rebound Tenderness	28 (28.3%)	61 (32.8%)	0.43
Ultrasound (direct signs)	46 (46.5%)	99 (53.2%)	0.28
WBC(109/L)	15.3 ± 3.5	15.6 ± 4.1	0.52
PCT(ng/mL, normal value: 0–0.5)	7.9 ± 3.6	7.5 ± 3.5	0.37
CRP (mg/L, normal value: 0–8)	22.6 ± 5.7	22.8 ± 5.6	0.78
Albumin (g/L, normal range, 35–50)	32.4 ± 8.6	31.8 ± 8.7	0.58
PLR (>188.5), *n* (%)	220.98 ± 143.73	172.14 ± 88.39	0.13
LCR (<0.121), *n* (%)	0.109 ± 0.093	0.112 ± 0.101	0.81
NLR	6.89 ± 5.22	5.15 ± 4.01	0.22

PLR, platelet-to-lymphocyte ratio; LCR, lymphocyte–C-reactive protein ratio; NLR, neutrophil–lymphocyte ratio.

Several inter-group differences in clinical manifestations were noted. While the rates of migratory abdominal pain (*P* = 0.055) and nausea/vomiting were comparable, right-lower-quadrant tenderness occurred less commonly among pregnant patients (*P* = 0.046). No inter-group differences were found for admission CRP and WBC levels.

The sensitivity of abdominal ultrasound was 46.5% in the pregnant group and 53.2% in the non-pregnant group. All patients underwent ultrasound and in the subgroup of patients (*n* = 32) with inconclusive ultrasound but persistent clinical suspicion, MRI demonstrated 100% concordance with final pathological findings. Time from admission to surgery was not significantly different between groups.

## Intraoperative and postoperative characteristics

Intraoperative and postoperative results are shown in Table 2. Operative time was similar between groups; conversion to open surgery was more frequent in pregnant women (*P* < 0.001); postoperative hospital stay was longer (5.6 ± 1.7 vs. 4.4 ± 1.1 days, *P* = 0.0056). The rate of laparoscopic appendectomy decreased with advancing gestational age 100% (22/22) in the first trimester → 87.2% (41/47) in the second trimester → 70% (21/30) in the third trimester.

**Table 2:** Intraoperative and postoperative characteristics in the eligible population.

Characteristics	Pregnant *n* = 99	Non pregnant *n* = 186	*p* Values
Operative time	49.4 ± 16.7	53.8 ± 15.9	0.32
Conversion to open surgery	15 (15.2%)	6 (0.032%)	<0.001
Estimated blood loss	10.2 ± 3.9	10.9 ± 3.8	0.17
Complicated appendicitis	28 (28.3%)	68 (36.6%)	0.16
Negative appendectomy	14 (14.1%)	18 (9.7%)	<0.001
WBC(109/L) on POD 5	8.8 ± 3.9	9.2 ± 3.7	0.41
PCT(ng/mL, normal value: 0–0.5) on POD 5	1.8 ± 0.7	1.7 ± 0.6	0.23
CRP (mg/L, normal value: 0–8) on POD 5	9.4 ± 3.8	9.5 ± 4.2	0.84
Postoperative intra-abdominal abscess	16 (16.2%)	36 (19.4%)	0.51
Postoperative surgical wound infection	5 (5.1%)	22 (11.8%)	0.063
Microbiological positive, *n* (%)	18 (18.2%)	48 (25.8%)	0.15
C. difcle infection	1 (1.0%)	8 (4.3%)	0.17
MRSA infection	0 (0.0%)	3 (1.6%)	0.55
Diarrhea	22 (22.2%)	46 (24.7%)	0.64
Early ileus	27 (27.3%)	49 (26.3%)	0.87
Duration of IV antibiotics, days, Mean ± SD	5.1 ± 1.3	6.5 ± 0.6	0.013
Length of hospitalizations	5.6 ± 1.7	4.4 ± 1.1	0.0056

Within 30 days after appendectomy, 2 pregnant women underwent cesarean section, classified as Clavien–Dindo ≥3 complications one 29-week patient underwent emergency cesarean section on postoperative day 1 due to abnormal fetal heart rate monitoring; one 31-week patient was readmitted 3 weeks postoperatively for suspected chorioamnionitis and underwent cesarean section. No other severe maternal complications occurred. One 9-week patient had a miscarriage on postoperative day 1 after appendectomy for suppurative gangrenous appendicitis; this event was not classified as a maternal complication. The histologically negative appendectomies was 14.1% in the pregnant group and 9.7% in the non-pregnant group (*P* < 0.001).

### Follow-up and adhesive small bowel obstruction (ASBO)

Median follow-up was 31 months (range 12–67 months). During follow-up, 15 of 99 patients (15.2%) in the pregnant group developed obstruction events, and 22 of 186 patients (11.8%) in the non-pregnant emergency appendectomy group, with no significant difference. Three patients (0.8%) in the non-pregnant group required surgical intervention for suspected strangulation, 2 of whom were confirmed to have intestinal strangulation; no strangulation cases occurred in the pregnant group.

## Discussion

The diagnosis and management of acute appendicitis during pregnancy are extremely challenging for clinicians. To our knowledge, this is one of the relatively large-scale single-center retrospective studies focusing on appendectomy in pregnant patients within the 2020–2025 period.

Diagnosis is difficult because symptoms such as nausea, vomiting, and abdominal pain are difficult to distinguish from physiological discomfort of pregnancy (13). Diagnosis cannot be based solely on history and physical examination (14). This study found differences in some clinical signs between the two groups pregnant women had lower rates of right lower quadrant tenderness, abdominal muscle rigidity, and rebound tenderness. Classic acute appendicitis typically begins with periumbilical pain that migrates to the right lower quadrant as inflammation progresses; anorexia, nausea, and vomiting are common but have low discriminative value; right lower quadrant tenderness, muscle rigidity, and rebound tenderness have higher predictive value. Previous studies have noted that pregnant patients less often present with classic findings, especially in the second and third trimesters, when pain location may be atypical. Few studies have analyzed these signs in depth, but a recent report also found lower right lower quadrant tenderness in pregnant women (15, 16). Symptom duration 24 hours is usually associated with a lower probability of appendicitis, yet in this study, pregnant women presented significantly later (24 hours after symptom onset) than non-pregnant women. These findings confirm the consensus that appendicitis in pregnancy presents atypically, supporting the need for a more comprehensive diagnostic workflow.

World Society of Emergency Surgery (WSES) guidelines state that acute appendicitis in pregnancy should not be diagnosed based on symptoms and signs alone; laboratory and serological inflammatory markers must be integrated (17). Historically, higher histologically negative appendectomiess in pregnancy were considered acceptable, but unnecessary surgery should be minimized due to associations with adverse neonatal outcomes. Although blood tests and imaging can aid diagnosis, imaging use is limited by potential fetal adverse effects and equipment availability. Delayed diagnosis of acute appendicitis increases maternal and fetal mortality and complication rates (18). Therefore, early and accurate diagnosis in pregnant women with suspected acute appendicitis is critical (19).

No specific laboratory markers exist for diagnosing acute appendicitis; WBC and CRP are the most commonly used inflammatory indicators. Even if CRP and WBC are helpful, mild leukocytosis in normal pregnancy complicates interpretation (20, 21). In this study, mean WBC was slightly higher in pregnant women (13.5 × 10⁹L vs. 12.5 × 10⁹L), inconsistent with some literature. Studies suggest WBC 18 × 10⁹L has high predictive value for appendicitis in pregnancy, which was confirmed here, with no significant difference in positive predictive value between groups (88.2% pregnant vs. 93.2% non-pregnant). CRP levels were similar between groups. Thus, the diagnostic value of inflammatory markers is comparable in pregnant and non-pregnant populations. Importantly, WBC 18 × 10⁹L or CRP 10 mgdL in pregnancy warrants high suspicion and is strongly associated with appendiceal perforation. Other markers including neutrophils, lymphocytes, RDW, MPV, platelets, NLR, and PLR have also been used for diagnosis (21). Yazar et al. reported significantly higher NLR and PLR in pregnant women with acute appendicitis than in healthy pregnant controls (13); Baskıran et al. found higher NLR and PLR in the second and third trimesters than in the first trimester among patients with acute appendicitis (7). Theilen et al. identified WBC 18 × 10⁹L as one of the most important diagnostic markers in pregnancy (22); another study showed higher WBC, neutrophils, NLR, and PLR in the acute appendicitis group than in the normal appendix group (23).

Preoperative imaging has significantly reduced histologically negative appendectomiess in female patients and is a key component of the diagnostic pathway (10). Consistent with previous literature, ultrasound was the first-line imaging modality for suspected appendicitis at our hospital, with utilization rates 97% in both groups (9). Reproductive-age women have a higher than previously recognized risk of radiation-induced malignancy, and CT was avoided in pregnancy; no pregnant patients underwent CT in this study. Ultrasound accuracy and sensitivity were significantly lower in pregnant women, creating diagnostic challenges (24). A recent meta-analysis confirmed MRI has 90% sensitivity and specificity for suspected appendicitis in pregnancy, making it an excellent choice. The relatively low overall MRI utilization rate in our cohort may reflect that standardized MRI diagnostic guidelines for gestational appendicitis were not universally popularized in clinical practice during 2020–2025, alongside limited after-hours on-demand MRI availability in our hospital. The 100% diagnostic accuracy in these 32 cases supports increased MRI use in the future. WSES guidelines recommend MRI when ultrasound is inconclusive in pregnancy. Short-term observation and repeat evaluation when diagnosis is uncertain can also reduce negative resection rates without increasing perforation risk.

Reported negative resection rates vary from 30% to 12.1% by Bazdar et al. Theilen et al. noted a negative resection rate as high as 33% even with MRI (25). Pedrosa et al. reported MRI is effective for excluding appendicitis in pregnancy but has low positive predictive value. Pedroza et al. found a high false-positive rate, with a 29% negative resection rate (26). A recent meta-analysis reported MRI diagnostic value of 96%, with sensitivity and specificity comparable or superior to CT (10). The negative resection rate was 13.08% in the control group in this study, with no significant difference between groups. More liberal use of MRI may explain why diagnostic challenges did not lead to higher misdiagnosis rates in pregnant women.

Regarding intraoperative features and postoperative outcomes, this study found no higher perforation rate in pregnant women (16% vs. 10%). Few studies have examined whether perforation risk increases in pregnancy, with conflicting conclusions. Atypical clinical presentation did not prolong time from admission to surgery in this study, so it cannot explain the higher perforation rate. Consistent with other studies, pregnant women had longer operative and hospital stays and higher CCI scores, but complications were mostly minor. Apart from 2 cesarean sections within 30 days postoperatively, no severe complications occurred in pregnant women. Consistent with reported literature (27, 28), laparoscopic appendectomy can be successfully performed in most pregnant women with appendicitis, with favorable prognosis. The maximum gestational age in this case series was 33 weeks. One patient at 32 weeks of gestation underwent conversion to open surgery due to technical difficulties during laparoscopy.

Limitations of this study include its retrospective design based on medical records, with potential for incomplete documentation, interpretation bias, and inconsistent assessment. The selective MRI examination leads to selection bias, so the MRI diagnostic performance conclusion is only applicable to the subgroup with indeterminate ultrasound results rather than all suspected appendicitis patients. The perfect diagnostic performance of MRI cannot be generalized to all pregnant populations with suspected acute appendicitis. Future prospective multi-center studies with larger MRI cohorts are still needed to verify the diagnostic efficacy of MRI. The 5-year study span with possible variations in clinical management; and variability in appendicitis diagnostic workflows across hospitals and countries, particularly in ultrasound use and scoring systems. Nonetheless, this study provides important evidence regarding clinical presentation, management strategies, and outcomes of appendicitis in pregnancy.

## Conclusion

Diagnosis of acute appendicitis during pregnancy is challenging for clinicians. This study confirms differences in clinical presentation between pregnant and non-pregnant women; MRI evaluation is associated with a lower proportion of histologically negative appendectomies in pregnant patients with suspected acute appendicitis in this cohort. It further emphasizes that laparoscopic appendectomy during pregnancy is safe and does not increase the risk of severe complications.

## Data Availability

The original contributions presented in the study are included in the article/supplementary material, further inquiries can be directed to the corresponding author/s.
